# A Fully Integrated Humidity Sensor System-on-Chip Fabricated by Micro-Stamping Technology

**DOI:** 10.3390/s120911592

**Published:** 2012-08-27

**Authors:** Che-Wei Huang, Yu-Jie Huang, Shey-Shi Lu, Chih-Ting Lin

**Affiliations:** Graduate Institute of Electronics Engineering, National Taiwan University, Taipei 10617, Taiwan; E-Mails: d97943030@ntu.edu.tw (C.-W.H.); molimo84@gmail.com (Y.-J.H.); sslu@ntu.edu.tw (S.-S.L.)

**Keywords:** humidity sensor chip, sensor system-on-chip, polyaniline, micro-stamping process

## Abstract

A fully integrated humidity sensor chip was designed, implemented, and tested. Utilizing the micro-stamping technology, the pseudo-3D sensor system-on-chip (SSoC) architecture can be implemented by stacking sensing materials directly on the top of a CMOS-fabricated chip. The fabricated sensor system-on-chip (2.28 mm × 2.48 mm) integrated a humidity sensor, an interface circuit, a digital controller, and an On-Off Keying (OOK) wireless transceiver. With low power consumption, *i.e.*, 750 μW without RF operation, the sensitivity of developed sensor chip was experimentally verified in the relative humidity (RH) range from 32% to 60%. The response time of the chip was also experimentally verified to be within 5 seconds from RH 36% to RH 64%. As a consequence, the implemented humidity SSoC paves the way toward the an ultra-small sensor system for various applications.

## 1. Introduction

With the advancement of communication and information technologies, sensor networks have become an emerging technology to be developed for the next generation [1]. This technology enables intelligent connections between groups of sensors and promotes the autonomous environment for future life [2]. In addition to traditional sensor requirements, such as sensitivity and selectivity, low-power consumption, *in-situ* data processing capability, and wireless communication are also essential sensor system characteristics to be implemented within the sensor network technology. To build ubiquitous sensor networks, moreover, the small system form factor should be addressed to seamlessly integrate the sensor system into environments. To match the above requirements, monolithic sensor architecture has been proposed and implemented by micro-electro-mechanical system (MEMS) technology for years [3,4]. However, most of previous works suffer from the chip area consumed by sensor devices, *i.e.*, sensing area is much larger than the circuit area. As a consequence, novel sensor chip architectures need to be developed.

Among the various sensing quantities sought in different applications, humidity is one of the most important factors [5–7]. As a result, different kinds of materials, such as ceramic [8] and semiconductor [9], have been investigated as humidity sensing materials. However, most of these humidity sensing materials are either complex to integrate with CMOS chip because of the elevated operational temperature or have high power consumption. This leads to the fact that traditional humidity sensors are not suitable to combine with emerging technologies, such as sensor networks or machine-to-machine systems. Therefore, polymer-based humidity sensors have attracted much attention because of their potential flexibility, simplicity of fabrication, and low cost [10–12]. Among the different kinds of polymer-based humidity sensing materials, polyaniline is one of the most promising candidates to achieve low cost and low power consumption humidity sensing devices [2]. In addition, our previous study demonstrated a solution-based polyaniline humidity sensing material with high sensitivity of 0.09 [LogΔI/ΔRH(%)], good selectivity in CH_4_, CO and CO_2_ environment, and good temperature stability of 7.85 × 10^−10^ A/°C [12]. In this work, as a consequence, a heterogeneous sensor system-on-chip for humidity sensing is proposed, designed, and implemented.

With the circuits fabricated by a CMOS process, specifically the Fe-Al-polyaniline humidity sensing material is micro-stamped on the top of CMOS circuits to form the pseudo three-dimensional (3D) sensor chip architecture. 3D integration is one of the methods to integrate heterogeneous layers with interconnecting metal lines to improve performance or create new functions of chips [13]. With this heterogeneous integration, the developed humidity sensing material can be integrated with CMOS circuits to meet the requirements of sensor network technologies. Based on this work, a fully functional sensor system-on-chip for humidity sensing in future sensor network applications is implemented and experimentally demonstrated.

## 2. Experimental Section

### 2.1. Material and Sensing Mechanism

**Figure 1.**
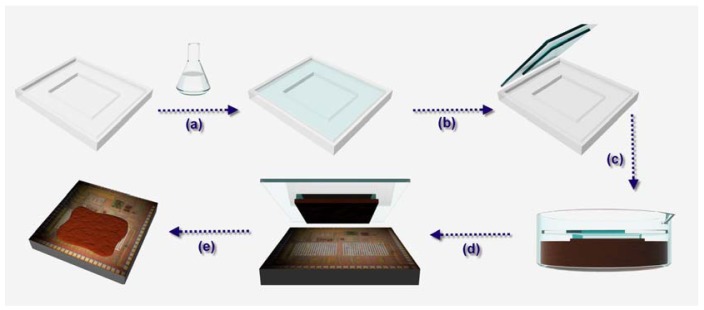
The schematic of the developed sensing film transferred onto CMOS chip: (**a**) PDMS is poured on a polymethylmethacrylate (PMMA) structure; (**b**) PDMS is cured and peeled off the PMMA structure; (**c**) The PDMS is cut for suitable size; (**d**) The cut PDMS is immersed in the synthesized polyaniline blends; (**e**) and (**f**) Polyaniline blends is stamped on interdigitated electrodes which are on the top of the analog circuit to form humidity sensor system-on-chip.

To fabricate the sensing material, a solution-based process has been developed [12]. In brief, polyaniline is chemically synthesized by copolymerization of aniline (ACROS 99%) and formaldehyde (ACROS 37%) in 11 mL hydrochloric acid solution (10 M). Then to the solution ferric chloride (ACROS 98%) and aluminum chloride (ACROS 99%) solutions are added with a volume ratio of 1:2 to form a well-mixed solution. This is the critical step because the metal ions, Fe^3+^ and Al^3+^ with specific concentration, can affect the selectivity and resistance of the blends. Following stirring within 200 mL 10% NaOH solution and precipitation, polyaniline blends can be obtained. Subsequently, the precipitate is washed with distilled water to reduce the acidity [14,15]. The sensing film can be formed by a micro-stamping method to transfer the suspended precipitation onto interdigitated electrodes on the top of the analog circuit to form the humidity sensor. The stamp is formed by polydimethylsiloxane (PDMS) which is poured on a pre-designed structure. A schematic of the copolymerization of aniline and formaldehyde and the key process of micro-stamping are shown in Figure 1.

The fundamental sensing mechanism of the polyaniline film has been investigated in previous works [16]. In brief, the acid-base reaction, NH_2_^+^ +H_2_O → NH + H_3_O^+^, is stimulated as the sensing film is exposed to humidity. As a result, the resistance of the polyaniline blends decrease. This reaction can be triggered in room temperature. The resistance change of polyaniline blends introduced by the reaction between low and high relative humidity (35% to 70% RH) is three orders [12]. The interface analog circuit can be used to measure the resistance change to estimate the relative humidity in the environment.

### 2.2. Circuit System Architecture

**Figure 2.**
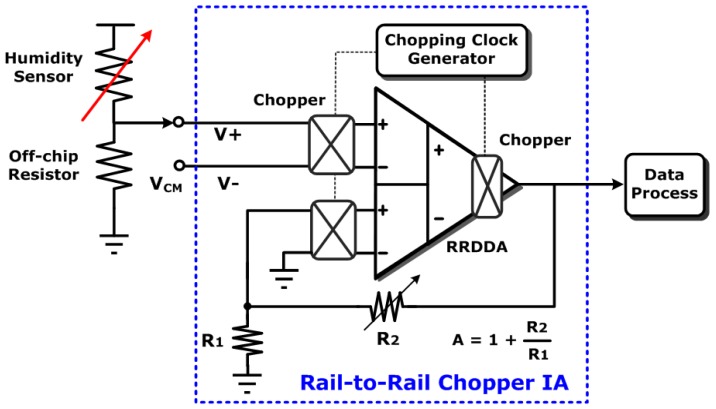
The schematic of analog front-end (AFE) readout circuit.

To the analog front-end (AFE) circuit, the humidity sensing device plays as a variable resistance in the voltage dividing circuit, as shown in Figure 2. Since the resistance of the sensing device is about 10^6^ to 10^8^ [12], the voltage dividing circuit is constructed of an off-chip resistor (10 MΩ) connected to it. According to the schematic shown in Figure 2, the resistance of humidity sensor will decrease as the relative humidity increases. As a result, the voltage across the sensing device will also decrease and the output voltage of AFE will become larger than previous values. Within AFE, a chopper differential difference amplifier (DDA) with features of low noise, high CMRR, and rail-to-rail input range to improve detection quality [17].

**Figure 3.**
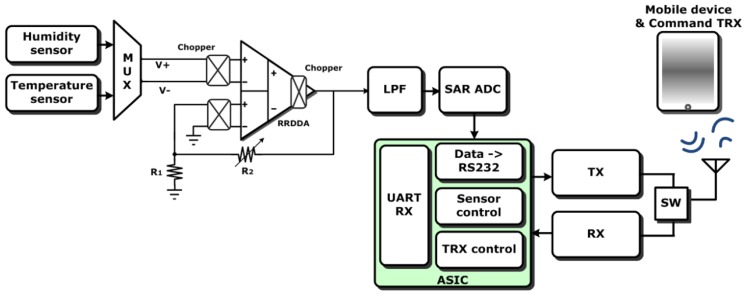
The block diagram of the developed humidity sensor system-on-chip (SSoC).

After the sensing is amplified, the sensing signal is then translated into digital data by a 10-bit successive approximation register analog-to-digital converter SAR ADC with low power consumption. With the help of a built-in digital signal processor (8051-based), this digital data is packaged into RS232 format to be sent by OOK wireless circuits (TX/RX) to an external host, *i.e.*, a cell phone or laptop, for further data analysis and saving. In addition, the wireless circuit cannot only transmit data but also receive command codes to wake up this SSoC from standby mode to active operation mode. Moreover, this chip also includes a built-in junction-based temperature sensor with good linearity. This temperature sensor shares the same chopper DDA with the humidity sensor by a multiplexer to reduce the circuit power consumption and complexity. Although the developed polyaniline blends have good temperature stability (7.85 × 10^−10^ A/°C) [12], this temperature sensor is integrated to calibrate the temperature information to compensate the whole SSoC operation. The block diagram of this humidity sensing SoC is shown in Figure 3.

### 2.3. Fabrication of the Pseudo 3D Heterogeneous Humidity SSoC

**Figure 4.**
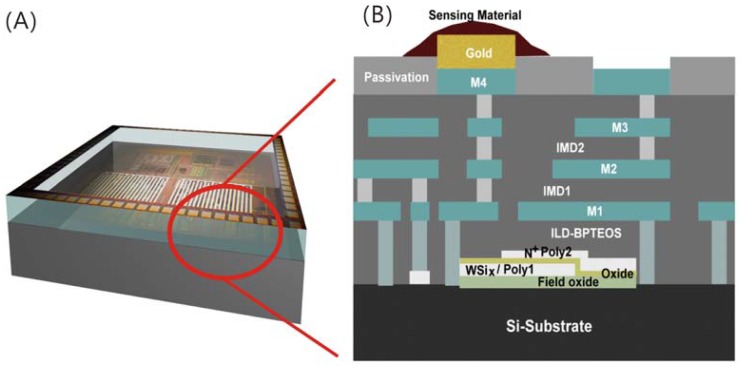
(**A**) The schematic of the developed humidity sensor system-on-chip (SSoC). The contact pads and interdigitated electrodes are at the top metal layer. And the analog/digital circuits are underneath the interdigitated electrodes; (**B**) The cross-section schematic of the developed humidity SSoC. The circuit elements, such as metals, dielectrics, and transistors, are under the micro-stamped sensing material.

To implement the proposed system architecture, the Taiwan Semiconductor Manufacturing Company (TSMC) 0.35 μm two-polysilicon-four-metal (2P4M) CMOS process is employed to implement the humidity sensor system. Previously designed analog and digital circuits are implemented following a standard CMOS process flow. The top metal layer (Metal 4) of the chip is used as the interdigitated electrodes. These interdigitated electrodes are directly connected to the AFE input by vias. It should be noted that the position of the intedigitated electrodes is on the top of the chopper DDA and OOK TX/RX to form the proposed pseudo-3D architecture. On the top of interdigitated electrodes, a layer of gold is e-beam evaporated to form a good contact to the sensing material [18]. Then the sensing material is coated on the top of electrodes by micro-stamping technology. The schematic of the fabricated SSoC can be shown in Figure 4. In the fabricated SSoC, the sensing device is stacking on the top of CMOS circuits. This not only reduces half of the chip area required by sensing devices but also decreases the interconnected metal line (signal line) between the sensing device and AFE. These improvements can reduce the cost and increase the signal-to-noise (S/N) ratio of the developed SSoC.

### 2.4. Experimental Protocol of Humidity SSoC Characterization

**Figure 5.**
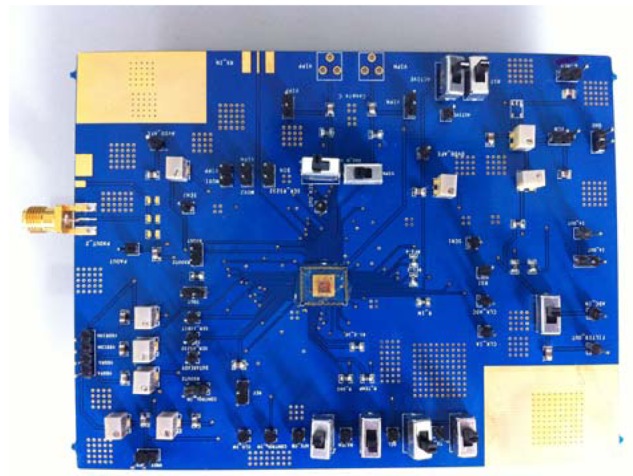
The picture of testing board module including the SSoC at the center.

The humidity sensing experiment was designed and used to determine the performance of the developed heterogeneous humidity sensing SoC. The testing board module can be seen in Figure 5. The schematic of our experimental setup was shown in our previous work [12]. The size of the testing chamber is 30 cm in length, 20 cm in width, and 15cm in height. To demonstrate the characterization of the developed SSoC, the chamber was pumped down by a mechanical pump and purged with compressed air three times. After purging, the moisture generated by boiling water was injected into the chamber. Then the signal from the SSoC was recorded for ten minutes. The chamber was pumped down after recording the signal. Following the same procedure, then another moisturized air sample was injected and the experimental signal was measured.

## 3. Results and Discussion

**Figure 6.**
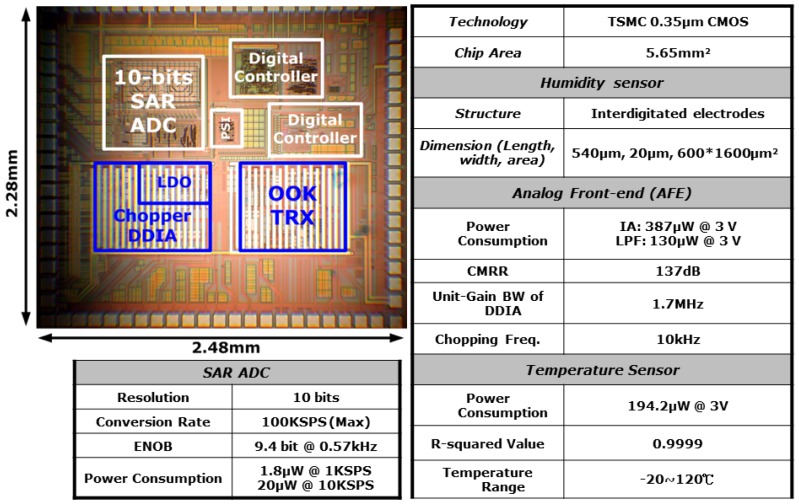
Chip photo and performance table of humidity sensing SoC.

The fabricated sensor chip photo and electrical performance are shown in Figure 6. The size of the chip is 5.65 mm^2^. The performance table shows the power consumption of the circuit system is around 730 μW without RF operation. By considering the sensing device and the off-chip resistor operation, the total power consumption is around 750 μW. The region marked by a blue line is the place for stamping the sensing material. After stamping the sensing material, the chip is wire-bonded and ready for test.

**Figure 7.**
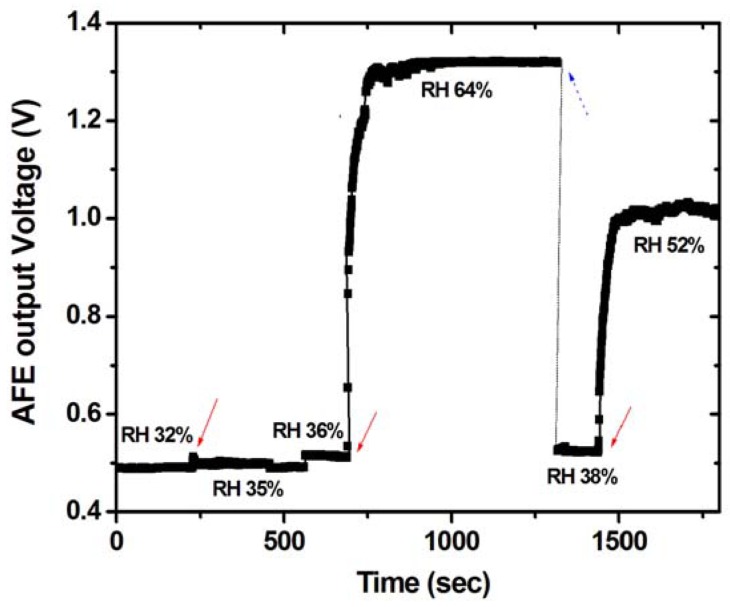
The response time test result. The initial relative humidity (RH) of experiment is about 36%. Then injecting the water vapor (ON state), the relative humidity becomes 64%. After about 600 sec, to remove the vapor (OFF state) the voltage signal falls to its initial value rapidly in room temperature.

**Figure 8.**
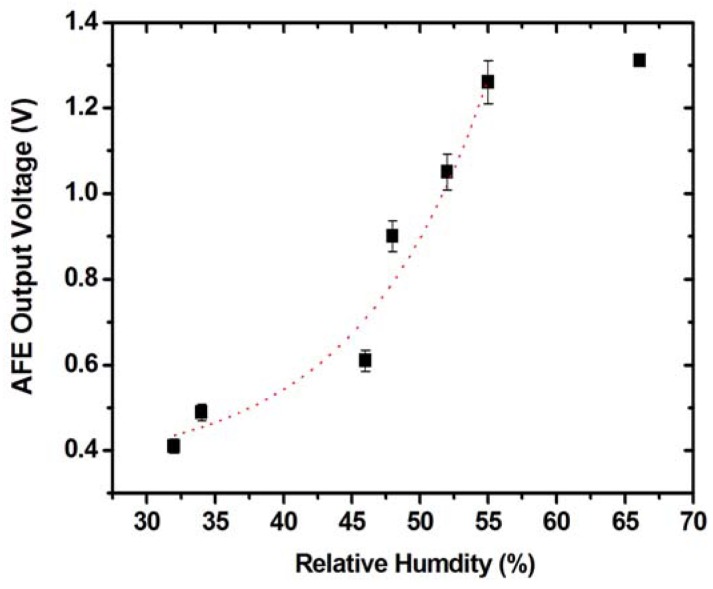
The sensitivity of the developed humidity SSoC. In this figure, data shows the mean ± standard deviation of the experimental measurement. Since the result after digital signal process is the same as the AFE output, it should be noted that this result shows the relationship between AFE output voltage and RH.

To examine if the sensing material is functional on the top of the interdigitated electrodes on the fabricated chip, the humidity SSoC was placed into a controlled chamber. With gating the injection of different relative humidity into the chamber, the voltage signal at the output of AFE was measured by Keithley 2400 source-meter. The experimental date of a single humidity injection can be shown in Figure 7. After pumping out the air and refilling the chamber with compressed air, the initial RH was 32% which was measured by a commercialized humidity sensor (Type 5332, WISEWIND, Taiwan). The slightly drift of the readout results from the leakage of valves. It can be noted that the AFE measured signal followed with the measurement of the commercialized humidity sensor. With the injection, the RH was increased to 64%. Following another pumping process and refilling with compressed air, the final RH in the chamber was back to 38%. It is clear that the voltage output of AFE followed these procedures. To estimate the response time of the SSoC, the time period between 20% to 80% voltage change is measured. It demonstrates a good time response, within 5 seconds, and reproducibility of the developed SSoC. In addition, the whole sensing process can be achieved at room temperature. Compared with other humidity sensors with several mWs power consumption and tens of seconds response time [19], the developed SSoC demonstrated the capability to be a practical humidity sensor system with low power consumption and fast time response. Furthermore, the sensitivity of the developed humidity SSoC was examined by similar procedures. The chip was exposed to different RH conditions, *i.e.*, from 32% to 66% in controlled chamber. The experimental results are shown in Figure 8. The measured results revealed that the AFE output voltage at RH32% is about 0.4 V and the voltage signal increased to 1.3 V at 66%. Because of the limitations of AFE circuit, the voltage output was saturated after 60%. It can be noted that the sensitivity was smaller than in our previous works [12]. This is due to the smaller sensing area (on the hundreds of μm scale) compared with the previous work (on a few cm scale) and the read-out circuit design to accommodate exponential sensing behavior into linear circuit operations. The interface circuit can be further improved to accommodate different ranges of RH changes. Nevertheless, the demonstrated sensing characteristic ranged from RH 32% to 55% was suitable for use in daily life. In addition, the built-in data processing and TX/RX promote the capabilities of the developed SSoC.

## 4. Conclusions

In this work, a fully-integrated humidity sensor system-on-chip (SSoC) is demonstrated. The SSoC is fabricated by the TSMC 0.35 2P4M CMOS process. Implemented by a micro-stamping technique to stack the sensing device on the top of CMOS circuits, the SSoC can be formed in a pseudo 3D architecture to obtain an ultra-small form factor (5.65 mm^2^). With low power consumption, *i.e.*, 750 μW without RF operation, experimental results have shown that the humidity sensing SoC had a fast response time of about 5 seconds and high sensitivity of about 30 mV/% at room temperature. In addition, the SSoC integrates a polymer-based humidity sensing device, AFE, SAR ADC, 8051-based microcontroller, and OOK wireless TX/RX on a single chip. Therefore, this work demonstrates not only a practical humidity sensor system-on-chip, but also a novel method to implement sensor systems for applications in sensor network technologies.
